# Mistaken Max befriends Duplo girl: No difference between a standard and an acted-out false belief task

**DOI:** 10.1016/j.jecp.2019.104756

**Published:** 2019-12-19

**Authors:** Beate Priewasser, Franziska Fowles, Katharina Schweller, Josef Perner

**Affiliations:** Centre for Cognitive Neuroscience, University of Salzburg, A-5020 Salzburg, Austria

**Keywords:** Theory of mind, Social cognition, Cognitive development, False belief understanding, Implicit false belief, Acted-out false belief tasks

## Abstract

With their Duplo task, Rubio-Fernández and Geurts (2013) challenged the assumption that children under 4 years of age cannot pass the standard false belief test. In an attempt to replicate this task on a sample of 73 children aged 32–51 months, we added a standard change of location false belief task as well as a Duplo true belief task. Performance on the latter is crucial for interpreting answers in the Duplo false belief task as to whether they reflect evidence for understanding or merely exhibit a difference in guessing rate. We found (a) a greater variability of response types in both Duplo tasks, (b) no evidence that responses in the Duplo tasks reveal earlier competence than those in the standard false belief test, and (c) a reassuring correlation between false belief tasks, suggesting that the Duplo task does pick up understanding of belief in light of the standard test.

## Introduction

The classical finding that the standard false belief test—also known as the “Maxi” task (Wimmer & Perner, 1983)—is passed by 4 years of age (Wellman, Cross, & Watson, 2001) has been challenged by the use of indirect indicators such as anticipatory looking (Clements & Perner, 1994; Southgate, Senju, & Csibra, 2007), looking time (Onishi & Baillargeon, 2005), and neural activity (Southgate & Vernetti, 2014; Kampis, Parise, Csibra, & Kovács, 2015). Although the replicability of these findings with very young infants from 6 months to 2 years has been questioned recently (Kulke & Rakoczy, 2018; see also special issue of *Cognitive Development* edited by Sabbagh & Paulus, 2018), the finding that correct anticipatory looking occurs just before children turn 3 years old has been replicated in seven of seven studies (Kulke & Rakoczy, 2018). This early evidence preceding verbal answers in the standard test has been attributed to an implicit understanding of belief (Perner & Clements, 2000). The justification for this claim resides in the finding that children show no knowledge of the agent’s belief in a direct test, but only in an indirect test, commonly seen as a criterion for implicit knowledge (Reingold & Merikle, 1993). When directly asked where a mistaken agent will go to get an object that was transferred without his or her knowledge, children answer with the object’s actual location when at the same time their eye gaze shows some awareness that the agent will go to where he or she thinks it is. Alternatively, the eye gaze might be evidence for explicit understanding that remains obscured by the test question due to processing limitations (Baillargeon, Scott, & He, 2010) or misleading pragmatics (Helming, Strickland, & Jacob, 2016). Findings from Rubio-Fernández and Geurts (2013, 2016) provide potentially important evidence for resolving this controversy. Their claim is that by lowering processing demands, children become able to respond correctly to a verbal command, which is deemed possible only with explicit knowledge.

In Rubio-Fernández and Geurts’s (2013) Duplo false belief (DFB) task, 3- and 4-year-olds are told about a girl who stores bananas in one of two boxes. While she is turning away, the bananas are transferred to the other box, the same as in the standard false belief (SFB) story except for the following four differences. First, the girl never went out of sight; she merely stepped to the side where she could not see the manipulation. Second, the experimenter transferred the bananas instead of introducing an additional story character. Third and fourth, when the girl returned, children were *not* told what she wanted and were *not* asked a question; they were simply requested to finish the story operating the girl doll themselves. This was to avoid any need to inhibit knowledge of the bananas’ real location, which is thought to be particular difficult in the SFB task (Baillargeon et al., 2010; Leslie, 1994; Setoh, Scott, & Baillargeon, 2016). Mention of the girl’s desire to get the bananas also creates a strong pull towards their real location because the girl wants to go *where they are* and not *where she thinks they are* (Perner, Rendl, & Garnham, 2007).

Omitting the girl’s desire is, however, a risky undertaking. Without knowing what she wants, children may make her do anything, for example, go to the now empty box to put something else in there. They will make her go to where she believes the bananas are only if they assume that she wants to get the bananas. Because there is no indication of children making this assumption, it is essential that a Duplo true belief (DTB) control task is administered.

Rubio-Fernández and Geurts’ (2013, 2016) findings were impressive. Nearly all children (mean age of around 3½ years) made the doll go to the full box in the DTB condition (91% in Experiment 1 of the 2013 study^1^ and 100% in Experiment 1 of the 2016 study). In contrast, 80% of children made the doll go to the empty box in the DFB condition, whereas only about 22% gave the correct answer to the SFB question (deceptive content task in Experiment 1 of the 2013 study).

The data have important consequences for theories explaining early sensitivity to beliefs. First, the fact that children move the doll to the believed location of the bananas as early as they show anticipatory looking in the false belief task (Clements & Perner, 1994) speaks against an implicit knowledge of belief because intentional actions are typically carried out consciously. Second, the data question the explanation that the spontaneity of looking behavior accounts for earlier evidence over the elicited responses in the SFB task (Baillargeon et al., 2010) because children’s responses in the DFB task are also elicited. Third, the data speak against the pure processing load account by Baillargeon et al. (2010) that the SFB task requires children to understand the story plus the experimenter’s question. Although no specific question is asked, the experimenter nevertheless elicits a response with a range of more general questions.

The data also support some proposed explanations. First, the fact that the belief-related responses decline drastically with the mention of the bananas’ location or the girl’s desire (Rubio-Fernández & Geurts, 2013) goes well with the claim that the lure of reality cannot be suppressed (Setoh et al., 2016). Second, these effects could also be due to children’s difficulty in switching from their own perspective, enforced by mentioning the bananas’ location or the girl’s desire, back to the girl’s perspective (Rubio-Fernández & Geurts, 2013; Helming et al., 2016), as opposed to children needing to inhibit their representation of reality.

Given their theoretical importance, the Duplo results warrant a careful replication, especially given that the employed experimental design has some weaknesses to be ironed out. In particular, there was only one experiment in which children were randomly assigned to the DFB and DTB conditions (Rubio-Fernández & Geurts, 2016, Experiment 1). Unfortunately, no SFB task was administered to the same sample. The claim that 80% of belief-directed answers at 3 years 7 months of age could be made only by comparing them with performance on the SFB test from a different, slightly younger sample (3 years 5 months) in Rubio-Fernández and Geurts (2013). Arguing for a performance difference purely on age is risky given that the large meta-analysis of SFB tests by Wellman et al. (2001) suggests a non-negligible number of samples in the age range of 3 years 3 months to 4 years that showed a mean correct answer rate of 80% or higher (see Fig. 2 in Wellman et al., 2001).

Existing replications are either conceptual (Białecka-Pikul, Kosno, Białek, & Szpak, 2019; Dörrenberg, Wenzel, Proft, Rakoczy, & Liszkowski, 2019; Rubio-Fernández & Geurts, 2016) or did not include a true belief control (Kammermeier & Paulus, 2018). Without a DTB task, Kammermeier and Paulus (2018) could not decide whether the approximately 20% more frequent correct DFB answers compared to SFB answers reflected earlier evidence from the Duplo task or a difference in guessing rate. This in turn led to an exchange of opinions (Paulus & Kammermeier, 2018; Rubio-Fernández, 2018) as to what the baseline performance of children who do not understand belief might be. We argue that this discussion can be settled only with an experimental inclusion of the DTB control because it ensures that children ascribe the intended goal—of getting the bananas—to the agent. The current study, therefore, is the first to combine a direct replication method with a true belief condition. On the group level, the DTB task allows us to see whether the majority of children intuitively do ascribe to this goal. However, if this is not as cogent as the data of Rubio-Fernández and Geurts (2013, 2016) suggest, we cannot assume that every empty box response in the DFB task reliably indicates false belief understanding. In this case, DFB responses should be assessed in relation to DTB responses on the individual level. Consequently, before investigating the interesting specific effects claimed to be at work, we decided to replicate the study in a within-participants design including the DFB, DTB, and SFB tasks. This has the advantage of getting a more precise estimate of understanding if children give the correct answers to the DFB task as well as the DTB task. Rather than differences from chance level, we took the DTB condition as the baseline for the interpretation of DFB performance. To get the DTB condition involved, we relied on the joint probability between correct responses on the DTB and DFB tasks. Hence, if the DFB task reliably detects false belief understanding earlier than the SFB task, we should find (a) a significant performance difference between DFB × DTB and SFB (at the group level, between participants) or (b) significantly better performance in the DFB task than in the SFB task in the subgroup of DTB passers (at the individual level, within participants). In addition, the design allowed us to see whether the DFB performance correlates with that of the SFB task.

## Method

### Participants

Rubio-Fernández and Geurts (2013) tested whether the percentage of correct responses was above chance of 50%. They found a large effect size (Cohen’s *h* = 64) for their sample of *n* = 25. On this basis, we computed the necessary sample size for obtaining 80% power following the standard recommendation outlined by Cohen (1988) and implemented in the *pwr* package by Champely (2013). Although this calculation indicated a sample size of *n* = 15, we decided to test 75 children for the following reasons. First, it is inadvisable to use fewer participants for replications than in the original studies (Etz & Vandekerckhove, 2016). Second, to allow comparisons among the three tasks in case children needed to be excluded due to transfer effects of the within-participants design.

A total of 77 children from six nursery schools and two recreational facilities (Toy Museum and Indoor-playground) in the city of Salzburg, Austria, volunteered for this study. Of this sample, 4 children needed to be excluded because 2 children were not cooperative, 1 child had comprehension problems, and 1 child had an inaccurate date of birth given to the experimenter (after correction, it turned out that the child was too old for the sample). The final sample consisted of 73 children (28 girls) aged 32–51 months with a mean age of 41.92 months (*SD* = 4.87). Parents gave written consent, and if they agreed the session was recorded. Children received a small toy for participating.

### Design

Children participated in a DFB task, a DTB task, and a change-of-location SFB task. The order of tasks was counterbalanced with Latin square. To ensure a direct replication of both the DFB and DTB tasks (Rubio-Fernández & Geurts, 2013), the original banana story was always used as the first Duplo task,^2^ and to allow for a within-participants comparison, a parallel version (toy car story) was developed. Whereas color and position of the containers was kept constant, the direction of the objects transfer was counterbalanced.

### Procedure and materials

Children were tested by a female experimenter in a separate room in the day-care center or recreational facility. They sat at a table with the children sitting at a right angle to the experimenter, and each session lasted about 10 min. A total of 30 children were accompanied by a parent (*n* = 18), an older sibling (*n* = 3), or an educator (*n* = 9). Caregivers were seated in a chair some distance behind the children and were carefully instructed not to interrupt the testing procedure. To avoid procedural differences between our tasks and those of Rubio-Fernández and Geurts (2013, 2016), Paula Rubio-Fernández kindly sent us her comments on a videotape of each condition.^3^ Her recommendations were implemented in our final versions; see detailed instructions in Appendix A and videotapes of the procedure in OSF (Open Science Framework), https://osf.io/w2meu/.

### Duplo tasks

All props used in the two parallel versions of the tasks were Duplo toys. The main protagonist was a figure (boy or girl) of approximately 6 cm in size. For the containers, we used either two pink boxes (6. 4 × 6 × 3 cm) with a door (blue and yellow) or two treasure chests (5.5 × 4 × 4 cm; green and brown). The transferred object was a bunch of bananas (4 cm) or a toy car (3.3 cm). At the beginning of the experiment, children were allowed to explore the toys used in the specific task. After a while, the experimenter suggested acting out a story with the toys. She then placed the containers approximately 20 cm apart from each other on the table, facing the child. The approximate distance between containers and the child was 30 - 40 cm. Lastly, the object was placed approximately 20 cm in front of the containers and 10–20 cm in front of the child.

### Introduction

In the original version of the Duplo task, the experimenter told the child about how the protagonist loved eating bananas and, because she already had eaten a banana today, she stored them in one of two fridges. In the parallel version, the protagonist was a boy who loved playing with a toy car, which he then stored in one of two toy boxes.

### False belief condition

After the object was put into the container, the experimenter explained that the protagonist now wanted to take a walk. The experimenter walked the Duplo figure to the opposite edge of the table, where it was then placed with its back to the containers. During the transfer of the object and when asking epistemic prompts [see (1) and (2) in the “Epstemic prompts” section below], the experimenter whispered and behaved conspiratorially; for example, she put her finger to her mouth and had a slight smirk on her face.

### True belief condition

After the object was put into the container, the experimenter placed the Duplo figure facing both containers. Next, the transfer of the object and the epistemic prompt question (2) were acted out neutrally and without any conspiratorial cues. Announcing that the protagonist now wanted to take a walk, the experimenter walked the Duplo figure to the opposite edge of the table and back to the scene.

### Epistemic prompts

To make the child attend to the epistemic states of the protagonist, the following questions were asked: (1) (before transfer) “Can she/he see me from there where she/he is standing?” and (2) (after transfer) “Did the girl/boy see what I did?” The DFB condition included both kinds of prompts, whereas the DTB condition included the after-transfer prompt only, and both questions were treated as prompts rather than as control questions. Thus, irrespective of what the child answered, the experimenter continued by saying “No, she/he cannot see me!” and “No, she/he did not see what I did!” in the false belief condition and “Yes, she/he saw what I did!” in the true belief condition.

### Prediction test

After the experimenter had the Duplo figure come back from the walk, she placed it in front of the two containers and asked the child, “Do you want to play with the girl/boy now? What will happen next?” If the child did not respond spontaneously, the experimenter encouraged the child: “You can take the girl/boy now if you want to. What will she/he do next?” If the child still did not respond, the experimenter repeated the question: “What will she/he do next?”^4^

### Traditional false belief task

A PowerPoint animation of the standard change-of-location false belief story (Wimmer & Perner, 1983) was presented on a tablet and narrated by the experimenter. In the protagonist’s absence, a toy was transferred to a new location and children were asked to predict where the protagonist, who had returned to continue playing with the toy, will look for the toy (*test question:* “Where will Maxi look for the ball first?”). Seven *control questions* were asked to make sure that children had understood relevant story facts (“Where did Maxi put the ball?”, “Where is the ball now?”, “Who placed it there?”, and “Where did Maxi place the ball in the beginning?”) and could still remember them at the end of the story (“Where is the ball now?”, “Where did Maxi place the ball in the beginning?” and “Did Maxi see that his sister put it there?”). If children were not able to answer a question correctly, the story was repeated and the correct answer was given.

## Results

For each task some children needed to be excluded due to experimenter errors (DFB: 1; SFB: 1), caregiver errors (DFB: 2), lack of cooperation by the child (DTB: 3; DFB: 2; SFB: 2), ambiguous responses to the test (DFB: 2; DTB: 2), starting to enact the false belief scenario in the DTB task (5), and failing on 50% or more of the control questions^5^ in the SFB (6). The number of children excluded in each task can be seen in Table 1 together with children’s answers to the epistemic prompts (DTB and DFB) and their responses to the tests.

### Confirmatory analysis

In both Duplo tasks, children had problems in giving answers or giving correct answers to the epistemic questions. Following Rubio-Fernández and Geurts (2013, 2016), we treated these as prompts and not as questions to be answered correctly. In the test, a surprisingly large number of children did not give any response, and several said “don’t know” or did something other than moving the doll to one of the boxes (e.g., let the protagonist go for a walk). In line with Rubio-Fernández and Geurts (2013), children who did not move the doll to one of the two locations (full/empty) in the DFB and DTB tasks but showed other responses are treated as exclusions; hence, success rates are relative to the sum of definite box responses (Σ±). For the confirmatory analysis, we first assess children’s test reactions against chance level (two-choice binomial test, hypothetical probability of success = .50, two-tailed) and then compare performance in the DFB and SFB tasks between and within participants.

In none of the tasks did performance differ from chance. Success rates in the DFB (*n* = 43), DTB (*n* = 37), and SFB (*n* = 64) tasks were 58% (*p* = .36), 62% (*p* = .188), and 41% (*p* = .169), respectively. For between-participants comparisons, we use children’s response to the first task administered (Table 2). A chi-square test with Yates correction revealed no significant difference in children’s performance on the DFB and the SFB tasks, *χ*^2^(1, *N* = 31) = 0.382, *p* = .458. A total of 39 children gave a valid (full or empty box) response in both the DFB and SFB tasks and, therefore, can be used for within-participants comparison (see left panel of Table 3). Neither on the individual level is there evidence for earlier understanding revealed by the Duplo test than by the standard test, exact McNemar, *χ*^2^(1) = 0.75, *p* = .387, odds ratio (OR) = 0.50, 95% confidence interval (CI) [0.11, 1.87].

### Additional analyses

#### Transmission effects

To assess these results properly, we need to make sure that our within-participants design did not work against the Duplo tasks because tasks presented earlier might have had a detrimental effect on Duplo tasks later in the series. There is, however, no sign of such an effect. The percentage of correct answers did not differ from first, to second, to third positions for any of the tasks, all *χ*^2^(2) ≤ 1.08, *p* ≥ .583, *Φ*_Cramer_ ≤ .13. The same held true for Duplo story context (banana or toy car), *χ*^2^(1) ≤ 2.74, *p* ≥ .098, *Φ*_Cramer_ ≤ .27,^6^ and for direction of transfer in the Duplo tasks (left → right or right → left), *χ*^2^(1) ≤ 0.18, *p* ≥ .668, *Φ*_Cramer_ ≤ .07.

In the original study (Rubio-Fernández & Geurts, 2013, Experiment 1), the DFB task always followed the SFB task. To control for a priming effect, we compare responses in the DFB task when it was administered immediately after the SFB task (*n* = 23) with when it was administered as the very first task (*n* = 20). There is no evidence for such an effect (DFB as first task: 7 empty, 4 full, and 9 other responses; DFB after SFB task: 11 empty, 8 full, and 4 other responses), *χ*^2^(2) = 3.96, *p* = .138, *Φ*_Cramer_ ≤ .30.

#### Extended baseline

Because there is no convincing argument for excluding from the analysis children who responded in an unexpected way (Kammermeier & Paulus, 2018), we also report the success rates relative to all responses (other responses also coded as incorrect) in Tables 1 and 2.

#### Analysis of the DFB task in light of the DTB task

As we argued, empty-box responses in the DFB task should be interpreted as evidence for understanding belief only if they correspond with full-box responses in the DTB task. Therefore, we compare the joint probability DFB × DTB with performance in the SFB task on both the group and individual levels.

For the between-participants analysis (first tasks only, strictly replicating the design of the original Duplo studies), false belief competence is reflected in the joint probability of correct DTB and DFB answers of .45 (7/10 × 7/11; see Table 2). The 95% confidence interval^7^ around this joint probability is ±.21^8^ [.24, .66]. The proportion of correct SFB answers of .45 is not significantly different; thus, the claim that the Duplo task provides earlier evidence of understanding belief than the SFB task is not supported.

The within-participants design allows us to identify the DTB × DFB response pattern of individual children shown in the right column of Table 3. Unfortunately, only 24 children responded with a definite box in both tasks, and only 6 children showed the desired response pattern. On these grounds Table 3 provides no evidence of any understanding because as many children showed the opposite pattern.

#### Correlations

Despite the fact that Duplo task performance did not outstrip SFB performance, there was a reassuring correlation with age in months for the DFB task, *r*_s_(43) = .39, *p* = .009, 95% CI [.102, .618], and for the SFB task, *r*_s_(64) = .34, *p* = .005, 95% CI [.103, .541]. No significant correlation was found for the DTB task, *r*_s_(37) = − .16, *p* = .301, 95% CI [−.46, .173]. Furthermore, the DFB and SFB tasks are significantly correlated, *r*_s_(39) = .39, *p* = .014, 95% CI [.085, .628], which suggests that the Duplo task does pick up understanding of belief in light of the standard test. When children start to understand the concept of belief, as mirrored in their SFB performance, the rationale of the DFB task also becomes comprehensible. For SFB passers, the narrative of the DFB task becomes less ambiguous given that 20 of 26 children (77%) responded with a definite box, whereas only 19 of 33 SFB non-passers (58%) did so.

## Discussion

There were two main findings: (1) greater variability of children’s test responses than originally reported and (2) no evidence of earlier competence in the Duplo procedure than in the SFB test.

We take the first of these findings to be a result of the open-ended nature of the Duplo task, leaving children with a wide field of interpretations, thereby making them susceptible to small environmental cues. In our sample, more than a third of children (35% in the DFB task and 41% in the DTB task) either said that they did not know what to do (5% and 13%, respectively), continued the story idiosyncratically (15% and 13%, respectively), or did not respond at all (15% and 16%, respectively). Response variability was also larger in Kammermeier and Paulus (2018) than in the original studies. Those authors argued that the problem is the open response format paired with a lack of control questions. This is likely given that we cannot be sure whether children understood the task as intended by the experimenter. For instance, in the Duplo task, children might assume that the Duplo girl wants to either look for her bananas or look for an empty box for something else. Dörrenberg et al. (2019) managed to make this apparently clearer with 94% and 85% correct answers for the DTB task.^9^ However, the more consistent performance in the DTB task did not produce earlier evidence for understanding belief over the SFB task in the DFB condition.

Another potential reason for the high variability in children’s reactions is that responding to the Duplo task requires children to switch perspectives.^10^ This feature distinguishes the Duplo task from other early false belief paradigms in which children take the same third-person perspective throughout the task. When asked to continue the story, they switch from being a third-person observer of the toy agent to being a first-person controller of that agent. The interpretation of responses is based on the supposition that children incorporate what they have learned about the agent (as a third-person observer) into their play (as a first-person actor). It is not explicitly controlled whether or to what extent children actually do this. Idiosyncratic and “don’t know” responses underline this problem, and there is no assurance that this does not also occur in empty-box and full-box responses. Hence, the switch between perspectives is another potential source for uncontrolled variability.

More important, assessing performance on the DFB, DTB, and SFB tasks on the same sample of children allows us to interpret children’s responses without needing to rely on the average age of different samples. Only children who gave correct responses in both the DFB and DTB tasks can be claimed to have some understanding of belief. However, there is no evidence on the individual level, nor is the joint probability of correct DTB and DFB answers significantly different from the proportion of correct SFB answers. Thus, there is no support for the claim that the Duplo task provides earlier evidence of understanding belief than the SFB task. In contrast, our joint probability is significantly lower than the corresponding values computed from the data reported by Rubio-Fernández and Geurts of .73 in 2013 and .80 in 2016. Both of them are clearly beyond the borders of the confidence interval (.24–.66). On these grounds, our data also fail to replicate the theoretically essential result of Rubio-Fernández and Geurts (2013, 2016); the data are significantly different from theirs and provide no evidence for their theoretical claim. The volatility of response rates highlights a potentially general problem of early false belief studies. In looking time and anticipatory looking paradigms, infants are not told a story, and thus—as in the Duplo task—it is not clearly communicated what the story agent wants. Children need to figure it out from the agent’s behavior in a few familiarization trials, which in many cases might not be sufficient. This might be one reason why the data from these techniques have been difficult to replicate.

## Figures and Tables

**Table 1:** Children’s reactions to tests and epistemic prompts/control questions per condition.

Task							Test reaction					Success rate (%)	
	Excluded	*N*	First prompt		Second prompt		No response	Don’t know	Other reaction	Full	Empty	Baseline^a^	
			No response	Incorrect	No response	Incorrect						Σ±	*n*
DFB	7	66	9	25	8	15	10	3	10	18^−^	25^+^	58	38
DTB	10	63	–	–	5	19	10	8	8	23^+^	14^−^	62	37
SFB	9	64	Control questions: 89.8% correct				0	0	0	38^−^	26^+^	41	41

*Note.* DFB, Duplo false belief; DTB, Duplo true belief; SFB, standard false belief; +, correct responses; −, incorrect responses; Σ±, sum of correct and incorrect responses.

a Success rates are reported relative to two different baselines: following Rubio-Fernández and Geurts (2013, 2016), using full and empty responses only (Σ±); and following Kammermeier and Paulus (2018), including all responses coded as incorrect (*n*).

**Table 2:** Children’s reactions to test questions in the first task administered.

First task	*N*	Test reaction			Success rate^a^ (%)	
		Other	Empty	Full	Σ±	*n*
DFB	20	9	7^+^	4^−^	64	35
DTB	22	12	3^−^	7^+^	70	32
SFB	20	0	9^+^	11^−^	45	45

*Note.* DFB, Duplo false belief; DTB, Duplo true belief; SFB, standard false belief; +, correct responses; −, incorrect responses; Σ±, sum of correct and incorrect responses.

a Success rates are reported relative to two different baselines: following Rubio-Fernández and Geurts (2013, 2016), including full and empty responses only (Σ±); and following Kammermeier and Paulus (2018), including all responses coded as incorrect (*n*).

**Table 3:** Contingencies of the Duplo false belief task with the standard false belief task and Duplo true belief task.

DFB task location	SFB task location		Total	DTB task location		Total
	Empty+	Full		Empty	Full+	
Empty+	16	8	24	5	6+	11
Full	4	11	15	6	7	13
Total	20+	19	39	11	13	24

*Note.* DFB, Duplo false belief; DTB, Duplo true belief; SFB, standard false belief; +, correct responses.

## Footnotes

1 Note that the true belief condition was added post hoc (*n* = 11).

2 Unfortunately, we could not follow our preregistered analysis plan regarding the direct replication of Rubio-Fernández and Geurts (2013). Due to an unexpectedly high number of children who did not move the puppet to a container, a between-participants comparison of the success rates in the first task was not possible. Of 20 children who had the DFB in the banana story context as their first task, only 11 moved the puppet to a container (7 chose the empty container). Of 22 children who had the DTB in the banana story context as their first task, 10 moved the puppet to a container (7 chose the container with the object).

3 Comments were based on two videos from an earlier student project in which the false belief condition was acted out in the original banana story, whereas the true belief condition was presented in a different story context (bed/doll story) than used in this study (car/toy-box story). However, all suggested comments could be applied in the new context.

4 For exploratory reasons, we asked children who responded by taking the puppet to one of the containers to explain their response: “Why did the girl/boy go there?” Not surprising for this age, children’s answers were not very informative and, therefore, were not analyzed.

5 For different reasons, only 53 children were asked all seven control questions; others were asked six questions (*n* = 6), five questions (*n* = 3), or four questions (*n* = 8). Six children answered 50% or less of the posed questions correctly. The remaining children answered on average 90% of the posed questions correctly (*M* = 89.8%).

6 There was a tendency for more correct answers in the original banana story version of the DTB condition, *χ*^2^(1) = 2.74, *p* = .098, *Φ*_Cramer_ = .27. In the DFB condition, no such tendency was found, *χ*^2^(1) = 0.72, *p* = .395, *Φ*_Cramer_ ≤ .13.

7 $\hat{p}\pm z\sqrt{\frac{p(1-p)}{n}}.$

8 $\hat{p}\pm 1.96\sqrt{\frac{.45(1-.45)}{21}}.$

9 They introduced a number of changes to the procedure to achieve greater uniformity across conditions—for example, including a warm-up procedure to familiarize children to the test response format, repeating the final prompt for test response up to three times if needed, repeating the story if epistemic prompts were answered incorrectly (only Göttinger subsample), verbally commenting that the object is changing location, phrasing one epistemic prompt question slightly differently, including conspiratory trickery in every condition, and introducing a second object.

10 We thank an anonymous reviewer for pointing out this argument.
